# Outcomes and influential factors in functional and dental rehabilitation following microvascular fibula flap reconstruction in the maxillomandibular region: a systematic review and meta-analysis

**DOI:** 10.1186/s40902-023-00392-8

**Published:** 2023-07-07

**Authors:** Elahe Tahmasebi, Elham Keykha, Samira Hajisadeghi, Hamidreza Moslemi, Shervin Shafiei, Mohammad Hosein Kalantar Motamedi, Arman Torabizadeh, Reza Tabrizi, Mostafa Alam

**Affiliations:** 1 Research Center for Prevention of Oral and Dental Diseases, Baqiyatallah University of Medical Sciences, Tehran, Iran; 2 School of Dentistry, Baqiyatallah University of Medical Sciences, Tehran, Iran; 3 Department of Oral and Maxillofacial Surgery, School of Dentistry, Shahid Beheshti University of Medical Sciences, Tehran, Iran; 4 Trauma Research Center, Baqiyatallah University of Medical Sciences, Tehran, Iran; 5 Private Practice, Tehran, Iran

**Keywords:** Free fibula graft, Microvascular graft, Implant success

## Abstract

**Background:**

This systematic review and meta-analysis aimed to evaluate the factors influencing and success rates of dental implants for functional and dental rehabilitation following microvascular fibula flap reconstruction in the maxillomandibular region.

**Main text:**

We conducted a comprehensive search of electronic databases, including MEDLINE, Web of Science, Embase, Scopus, and Cochrane’s CENTRAL, as well as gray literature sources and manual searches of notable journals. The search was performed from inception until February 2023. Studies were included if they examined functional and dental rehabilitation outcomes in patients receiving maxillofacial reconstruction using microvascular fibula flaps and were retrospective or prospective cohort studies involving human subjects. Case–control studies, research involving other reconstruction methods, and animal-based studies were excluded. Data was extracted and confirmed by two independent researchers, and risk of bias was assessed using the Newcastle–Ottawa Scale. Meta-analyses were conducted for dental implant and graft success rate, with separate analyses for different factors affecting the outcome. Heterogeneity was evaluated using Cochran’s Q test and the *I*^2^ test. The pooled success rate for implants was 92% and for grafts, 95%, with significant heterogeneity. Implants in fibular grafts had a 2.91 times higher failure rate than those in natural bones. Radiated bone and smoking were identified as factors influencing implant failure, with radiated bone having a 2.29 times higher risk and smokers having a 3.16 times higher risk compared to their respective counterparts. Patient-reported outcomes showed improvements in key areas such as dietary intake, mastication, speech, and esthetics. The success rates declined over time, emphasizing the importance of long-term follow-up.

**Conclusions:**

Dental implants in free fibula grafts generally have favorable success rates, with minimal bone resorption, manageable probing depths, and limited bleeding on probing. Implant success is influenced by factors such as smoking and radiated bone.

## Background

Maxillofacial defects can have a profound impact on a patient’s quality of life, affecting essential functions such as mastication, speech, articulation, and swallowing, as well as facial esthetics and symmetry [1]. These defects may result from various causes, including congenital abnormalities, traumatic injuries, or the surgical removal of tumors in the head and neck region [2]. The consequences of these defects extend beyond physical impairments, as they can also significantly impact a patient’s psychosocial well-being, leading to issues with self-esteem, social interaction, and overall mental health [1].

Several methods have been developed for the treatment and reconstruction of maxillofacial defects. These techniques include the use of autologous bone grafts, vascularized free flaps, and alloplastic materials [3, 4]. Autologous bone grafts, such as rib and tibia grafts or iliac crest and tibia grafts, can be combined with reconstruction plates for mandibular reconstruction [5]. Vascularized free flaps, particularly the free fibula flap (FFF), have gained popularity in recent years due to their potential for successful osseointegration and their ability to provide a robust and reliable source of bone for reconstruction [6]. Additionally, alloplastic materials, such as titanium plates and mesh, can be used to provide structural support and facilitate bone regeneration in cases where autologous grafts or flaps may not be suitable [7].

The choice of reconstructive technique is often determined by several factors, including the size and location of the defect, patient comorbidities, and the availability of donor tissue. A multidisciplinary team approach is crucial for achieving optimal outcomes in the management of maxillofacial defects, involving collaboration between oral and maxillofacial surgeons, prosthodontists, oncologists, and other healthcare professionals [8].

Following successful reconstruction of the mandible, dental rehabilitation is essential for restoring function and esthetics [9]. Dental implant systems offer numerous benefits, including the restoration of chewing ability, cosmetic appearance, jawbone preservation, and prevention of bone loss [10]. The integration of dental implants in reconstructed mandibles has been shown to provide satisfactory results [11–14].

Despite the advancements in reconstructive techniques and the potential benefits of dental implant systems, further research is needed to fully understand the long-term functional outcomes and dental rehabilitation success in patients who have undergone maxillofacial reconstruction using various techniques, including microvascular fibula flaps. By identifying best practices and potential areas for improvement, clinicians can continue to refine their approach to the management of maxillofacial defects, ultimately enhancing patients’ quality of life and overall well-being.

Therefore, in this systematic review and meta-analysis, we will examine the available literature, including randomized controlled trials, cohort studies, and case series, to assess the efficacy of dental implants and temporomandibular joint function in patients who have undergone maxillofacial reconstruction with FFFs. The primary outcome measures will include implant survival rates, implant-related complications, and functional outcomes, such as masticatory performance, speech intelligibility, and swallowing ability. Additionally, secondary outcome measures will focus on patients’ psychosocial well-being and quality of life.

Furthermore, we will explore potential factors that may influence the success rate of dental implants and temporomandibular joint function in patients with FFFs. These factors may include the type and extent of the mandibular defect, the timing of implant placement, patient demographics, and the presence of any comorbidities.

## Materials and methods

### Review strategy and study registration

Our systematic review and meta-analysis are conducted in accordance with the Cochrane Handbook Guidelines for Systematic Reviews of Interventions and the Preferred Reporting Items for Systematic Reviews and Meta-Analyses (PRISMA) statement [15].

### Focus question

The PICO framework for this investigation includes the following: the study population consisting of patients receiving maxillofacial reconstruction via microvascular fibula flaps; the intervention examining dental implant application and evaluation of temporomandibular joint functionality; comparisons made with alternative approaches to maxillofacial reconstruction, such as iliac crest or other grafts, and varying grafting time frames and patient comorbidities; and assessed outcomes, encompassing functional and esthetic results, and long-term implant and reconstruction stability and success.

### Information sources and search approach

We performed an exhaustive search of electronic databases, including MEDLINE, Web of Science, Embase, Scopus, and Cochrane’s CENTRAL, from their inception until February 2023. Additionally, we investigated gray literature sources like trial registrations, conference proceedings, and dissertations using search terms such as “maxillofacial reconstruction,” “fibula flap,” “dental implant,” and “temporomandibular joint function.” Manual searches of notable journals related to maxillofacial reconstruction with *IF* > 1 were also carried out. The search strategy was tailored for each specific database. A summary of the search strategies and the total number of studies retrieved are provided in Table 1.

**Table 1:** Custom search strategy for each database

Databases	Search strategy used	Hits
MEDLINE via PubMed	(microvascular OR composite flap OR microvascular transplant) AND (Fibula) AND (maxillofacial OR oral cavity)	395
Web of Science Core Collection	((ALL = (microvascular OR composite)) AND ALL = (fibula)) AND TS = (oral OR maxillofacial OR mandib? OR maxill?)	353
Embase	#1 'microvascularization'/exp OR 'microvascularization' OR 'composite flap'/exp OR 'composite flap' 3768 #2 ('fibula'/exp OR 'fibula' OR fibular) 24239 #3 'mouth cavity'/exp OR 'mouth cavity' OR 'maxillofacial injury'/exp OR 'maxillofacial injury' 109794 #4 #1 AND #2 AND #3	26
Scopus	ALL ( microvascular OR composite) AND TITLE-ABS-KEY ( fibula) AND TITLE-ABS-KEY ( maxillofacial AND injury OR maxillofacial AND trauma OR maxillofacial AND reconstruction)	287
Cochrane Central Register of Controlled Trials	#1 microvascular anastomosis 62 #2 microvascular 4444 #3 fibular 326 #4 maxillofacial 5248 #5 oral 239600 #6 (#1 OR #2) AND #3 AND (#4 OR #5)4	4
Total		1065

### Eligibility criteria and study selection

For this systematic review and meta-analysis, we established the following inclusion criteria: (1) studies examining the functional and dental rehabilitation outcomes in patients receiving maxillofacial reconstruction using microvascular fibula flaps, (2) retrospective or prospective cohort study designs, and (3) human subjects as the study population.

Exclusion criteria were defined as follows: (1) case–control studies, (2) research involving patients with other types of reconstruction methods not related to microvascular fibula flaps, and (3) animal-based studies.

Two review authors (H. M., S. S.) independently screened the titles and abstracts of search results to identify relevant studies, considering the PICO question and the established inclusion and exclusion criteria. Irrelevant studies were excluded from the review, and the rationale for their exclusion was documented. In cases of disagreement between the authors, a third author (A. T.) was consulted for resolution. The full texts of potentially relevant articles were further evaluated, with those not adhering to the PICO framework or the inclusion and exclusion criteria being eliminated and reasons for their exclusion provided.

### Data items and collection process

One researcher (H. M.) extracted data from the selected articles, while another researcher (S. S.) confirmed the accuracy of the data extraction. Information of interest included the study authors’ names, publication year, study type (retrospective or prospective cohort), the number of patients in treatment and control groups, patients’ average age, participants’ gender, study duration, inclusion and exclusion criteria, characteristics of fibula flap reconstruction, time intervals between defect and graft, the cause of the defect (congenital abnormalities, traumatic injuries, or surgical removal), dental implant properties, and outcomes such as implant stability and success, temporomandibular joint function evaluations, and functional and esthetic outcomes. This data was recorded using previously piloted forms. Table 2 contains a summary of the data related to the relevant studies.

**Table 2:** Summary of studies included in the review

**Author, year**	Study** design**	Number of** subjects**	Study** t**ime** frame**	Time between fibula graft and** implantation**	**Defect location**	**Implant type**	**Assessments**	Comorbidities:** radiation, malignancies, systematic disease**	**Indications for surgery**	**Number of implants in graft**	**Number of implants in natural bone**	Implant survival** rate**	**Graft survival rate**
**Ariga, 2017** [16]	Retrospective	10 (5 F, 5 M)	12 years (1998–2010)	Mean 13.4 months	6 in the maxilla and 27 in the mandible	Not specified	Clinical examination, radiological examination, interview using a questionnaire	N/A	Various, including ameloblastoma, fibrous dysplasia, ossifying fibroma, osteoradionecrosis, odontogenic myxoma, and central giant cell granuloma	33	N/A	100%	100%
**Attia, 2018** [17]	Retrospective	34 (11 F, 23 M)	11 years (2000–2011)	Dental implantation after 5 months	6 patients had maxillary defects, and 28 had mandibular defects	66 Xive, 45 BEGO, and 23 Synocta	Clinical assessments (dental status, oral condition, extent of prosthetic rehabilitation, postoperative complications, implant survival, and fibula transplant survival), radiological evaluations	Radiation (5 patients), malignancies (27 patients)	Tumor resection involving the jaw	134	53	91.05%	97%
**Bodard, 2008** [18]	Not explicitly mentioned in the text, but it appears to be a retrospective study	23 (6 F, 17 M)	NR	Mean delay between MFF and placement of implants was 23.5 months (8–60)	Mandible	43 KIIIW (NobelBiocare) and 32 CinyW (Serf)	Clinical examination, dentascan, postoperative clinical and radiographic controls, criteria for implant success and prosthetic success	14 patients (60.8%) underwent radiotherapy before reconstruction and 5 after (21.7%)	Mandibular osteoradionecrosis was the indication for reconstruction in 7 patients (30.4% of irradiated patients)	80	N/A	80%	NR
**Bodard, 2015** [19]	Retrospective	26 (9 F, 17 M)	NR	NR	Mandible	Not explicitly mentioned, but dimensions ranged from 3.75 × 10 mm to 4 × 15 mm	Number of osteotomies, number of implants, type of prosthesis, follow-up after prosthesis placement, preimplant surgery, quality of soft tissues, peri-implant complications, patient satisfaction (visual analog scale), esthetic outcomes, and improvement of masticatory function (questionnaire)	NR	NR	75	N/A	97.5%	NR
**Burgess, 2016** [20]	Retrospective	59 (24 F, 35 M)	6 years (2009–2015)	Mean time was 19 months (range, 0–141 months)	Head and neck neoplasia	Neoss, Straumann	Implant failure, adverse outcomes, implant survival by smoking status, implant survival by bone flap type	12 patients received radiation to the VBG before implant placement	Head and neck neoplasia	199	N/A	93.6% 5 years	NR
**Ch'ng, 2016** [21]	Retrospective	246 (80 F, 166 M)	6 years (2009–2015)	Mean time was 19 months (range, 0–141 months)	NR	AstraTec	Implant success, implant survival time, cumulative survival rates, risk factors for implant loss, completion of oral rehabilitation, odds ratios for osteoradionecrosis development	Preoperative radiation: 18 patients (7.3%) Postoperative radiation: 147 patients (59.8%) Chemotherapy: 99 patients (40.2%) Tobacco use (smoking): 102 patients (41.5%) Diabetes mellitus: 38 patients (15.5%)	NR	243	618 mandible, 271 maxilla	Mandible: 2.6% (16 out of 618) Maxilla: 2.2% (6 out of 271) Fibula: 8.2% (20 out of 243)	NR
**Chiapasco, 2006** [22]	Retrospective	59 (21 F, 38 M)	8 years (1995–2002)	3–12 months	Mandible or maxilla	20 ITI, 44 Nobel Biocare, 7 3i	Clinical and radiographic controls	NR	Tumors or osteoradionecrosis affecting the maxillo-mandibular complex	243	N/A	98.6%	94.9%
**De Santis, 2006** [23]	Retrospective	12	10 years (1993–2003)	Minimum of 6 months for osseointegration	Mandible	N/A	Clinical examination, radiographs, resonance frequency analysis (RFA) with Osstell	NR	Jawbone atrophy, cancer resection	76	N/A	100%	100%
**Gbara, 2007** [24]	Retrospective	52 initially, 30 followed up (18 M, 12 F)	2 years (1992–1994)	N/A	Mandible, maxilla	IMZ, ITI, Duraplant	Implant survival, mucositis, peri-implantitis	Radiation in 18 patients with malignant tumor	Ablative tumor treatment, jaw augmentation by severe atrophy, or osteomyelitis	117	N/A	97%	NR
**Goker, 2020** [25]	Retrospective	14 (8 F, 6 M)	5 years (2013–2018)	Mean interval period between two surgeries was 24.6 months (0 to 3.5 years)	Mandible, maxilla	10 Biomet 3i 2 Intra-Lock, 2 Megagen	Implant survival, graft survival, complications, patient characteristics, and implant survival	None	Tumors	40	16	85.6% (79.75% for implants in flaps, 100% in native bone)	85.7%
**Khadembaschi, 2020** [14]	Retrospective	100; 37 female (16 in FFF, 11 in DCIA, 8 in scapula, 2 in MFC) and 63 male (29 in FFF, 18 in DCIA, 14 in scapula, 1 in MFC, 1 in RFFF)	11 years (2008–2019)		Maxilla (11 in FFF, 10 in DCIA, 9 in scapula, 3 in MFC, 1 in RFFF), and mandible (34 in FFF, 19 in DCIA, 13 in scapula)	NS	Implant survival and success, prosthodontic success and failure	Radiation: RT to flap (15 in FFF, 4 in DCIA, 4 in scapula), RT pre-flap (9 in FFF, 1 in DCIA, 8 in scapula) Malignancies: malignant (35 in FFF, 9 in DCIA, 15 in scapula, 1 in RFFF) and nonmalignant (10 in FFF, 20 in DCIA, 7 in scapula, 3 in MFC)	Various pathologies	318 (150 in FFF, 98 in DCIA, 62 in scapula, 6 in MFC, 2 in RFFF)	N/A	1 year (93% FFF, 97.5% DCIA, 98% scapula, 100% MFC, 100% RFFF), 2 years (90% FFF, 97.5% DCIA, 98% scapula), 5 years (86% FFF, 89% DCIA, 93% scapula), 7 years (83% FFF, 80% DCIA, 93% scapula), 9 years (69% FFF)	NR
**Kniha, 2017** [26]	Retrospective	28 patients (14 with fibula flaps and 14 with DCIA flaps); 13 F, 15 M	3 years	6 to 9 months after reconstructive surgery	5 in the upper jaw, 23 in the lower jaw	Straumann, Camlog	Peri-implant bone resorption, implant survival rate, graft survival rate	10 patients (35.7%) treated with adjuvant radiotherapy before bony reconstruction	Malignant and benign tumors	109 implants (51 in DCIA flaps, 58 in fibula flaps)	N/A	98.3% for fibula flaps, 96.1% for DCIA flaps after 3 years	NR
**Lodders, 2021** [12]	Retrospective	23 (21 F, 23 M)	22 years (1995–2017)	NR	Mandibular and maxillary defects	Straumann	Implant survival, implant function, implant success, functional dental rehabilitation	Presurgical radiation	Tumor resection and immediate FFF reconstruction (primary-FFF) Tumor resection and delayed FFF reconstruction (secondary FFF) Resection for osteoradionecrosis (ORN) with immediate FFF reconstruction (ORN-FFF)	161	26 maxilla, 15 mandible	18.0% (29/161) in the FFF, 11.5% (3/26) in the native maxilla, 6.7% (1/15) in the native mandible	NR
**Lodders, 2022** [26]	Retrospective	57	11 years (2006–2017)	NR	Mandible	NS	EORTC QLQ-C30 and EORTC QLQ-H&N 35	NR	Patients diagnosed with head and neck cancer and had undergone maxillofacial reconstruction with a free fibula flap	55	N/A	NR	NR
**Menapace, 2018** [27]	Retrospective	23 (7 F, 16 M)	9 years (2006–2015)	NR	Mandible	NS	Graft survival, implant survival, oral competence, speech intelligibility, diet	NR	ORN or ON	121	N/A	92%	95% (21 of 22) FFTT survival rate
**Parbo, 2013** [13]	Retrospective	36 (13 F, 13 M)	13 years (1998–2011)	9.4 months	Partial mandibular resection, most frequently resected area was the lateral segment	Nobel Biocare, Astra, 3i, and Straumann	implant survival rate, graft survival rate	Radiation: 10 patients received presurgical radiotherapy; 16 patients received postsurgical radiotherapy Malignancies: most common diagnosis was squamous cell carcinoma, followed by sarcoma	Partial mandibular resection due to primary diagnosis (e.g., squamous cell carcinoma, sarcoma, or ameloblastoma) or secondary resection due to sequelae following the initial treatment of the primary pathology (e.g., osteoradionecrosis or osteomyelitis)	67	N/A	96%	97%
**Pellegrino, 2018** [28]	Retrospective	21 (6 F, 15 M)	17 years (1998–2015)	Mean of 20.8 months (range, 8–38 months)	Mandible and maxilla	25 Nobel System implants, 58 Friadent Dentsply, 8 BioHorizons, 6 Keystone, 6 BTK, 5 Biomet 3i	Implant survival, implant success, peri-implant mucositis, peri-implantitis, peri-implant bone loss, probing depth, and the presence of hyperplastic tissue surrounding the implants	Radiation, malignancies, systematic disease — 7 patients received adjuvant radiotherapy; 3 patients received postoperative chemotherapy without radiation therapy	Malignant and benign oral tumors	108	N/A	97.2% at 12-month follow-up, 86.5% at 60 months, 79.3% at 120 months	NR
**Zweifel, 2018** [29]	Prospective	8 patients (13 implants in trial group and 15 implants in control group)	2 years	N/A	Mandible	AstraTech, Straumann, Neoss OsseoSpeed	Postoperative computed tomography (CT) scans to compare the position of dental implants before and after surgery using reconstruction plate as a reference, measurements of distances, and angulations	NR	NR	13 implants in trial group and 15 implants in control group	N/A	N/A	NR

*Abbreviations*: *F* Female, *M* Male, *NR* Not reported, *NS* Not specified, *N/A* Not applicable

### Assessing risk of bias

Since most studies included in our review were retrospective or prospective cohort studies, we evaluated their risk of bias using the Newcastle–Ottawa scale (NOS) for cohort studies. This scale rates studies based on selection, comparability, and outcome assessment. The results of this assessment are tabulated in Table 3.

**Table 3:** The risk of bias of included studies based on the Newcastle–Ottawa scale

Newcastle–Ottawa criteria	Studies, year																	
	Ariga, 2017 [16]	Attia, 2018 [17]	Bodard, 2008 [18]	Bodard, 2015 [19]	Burgess, 2016 [20]	Ch'ng, 2014 [21]	Chiapasco, 2006 [22]	De Santis, 2006 [23]	Gbara, 2007 [24]	Goker, 2020 [25]	Khadembaschi, 2020 [14]	Kniha, 2017 [26]	Lodders, 2021 [12]	Lodders, 2022 [26]	Menapace, 2018 [27]	Parbo, 2013 [13]	Pellegrino, 2018 [28]	Zweifel, 2018 [29]
A. Selection (maximum of four stars)																		
1. Representativeness of the exposed cohort	★	★	★	★	★	★	★	★	★	★	★	★	★	★	★	★	★	★
2. Selection of the non-exposed cohort	☆	☆	☆	☆	★	★	☆	☆	☆	★	★	★	★	★	★	☆	★	★
3. Ascertainment of exposure	★	★	★	★	☆	★	★	★	★	★	☆	★	★	☆	★	★	★	★
4. Demonstration that outcome of interest was not present at start of study	★	★	★	★	★	★	★	★	★	★	★	★	★	★	★	★	★	★
B. Comparability (maximum of two stars)																		
1. Comparability of cohort on the basis of the design or analysis	☆☆	☆☆	☆☆	☆☆	★☆	★☆	☆☆	☆☆	☆☆	★☆	★☆	★☆	★☆	★☆	★☆	☆☆	★☆	★★
C. Outcome (maximum of three stars)																		
1. Assessment of outcome	★	★	★	★	★	★	★	☆	★	★	★	★	★	★	★	☆	★	★
2. Was follow-up long enough for outcomes to occur	★	★	★	★	★	☆	★	★	★	★	★	☆	★	★	☆	★	☆	★
3. Adequacy of follow-up of cohorts	★	★	★	★	★	★	★	★	★	★	★	★	★	★	★	★	★	★
Total (maximum of nine stars)	6	6	6	6	7	7	6	5	6	8	7	7	8	7	7	5	7	9

### Synthesis of the summary measures

The data from the chosen articles were considered suitable for meta-analysis if the therapeutic interventions were analogous and the outcomes were comparable. The pooled graft and implant success rate were performed by calculating the standard error for each study using the success rate and the number and then pooling the results. Also, the effects of different factors (like radiation and smoking) on the implant failure were calculated using risk ratio.

Separate meta-analyses were performed for dental implant outcomes and graft survival assessments, as well as different factors, to account for the diverse treatment approaches, comparison groups, and assessment timelines. Cochran’s Q test evaluated heterogeneity between studies, and the *I*^2^ test measured the extent of inconsistency in pooled calculations resulting from study heterogeneity. *I*^2^ values below 30% indicate low heterogeneity, values between 30 and 70% show moderate heterogeneity, and values above 70% represent significant heterogeneity.

Pooled implant and graft success rate were calculated using Stata 17 (StataCorp, TX, USA), and other analyses were performed using Review Manager 5.4 (Cochrane Collaboration, Denmark) software. A *p*-value of 0.05 was considered significant for hypothesis testing, while a *p*-value of 0.1 was employed for heterogeneity due to low power.

## Results

### Study selection

**Fig. 1:**
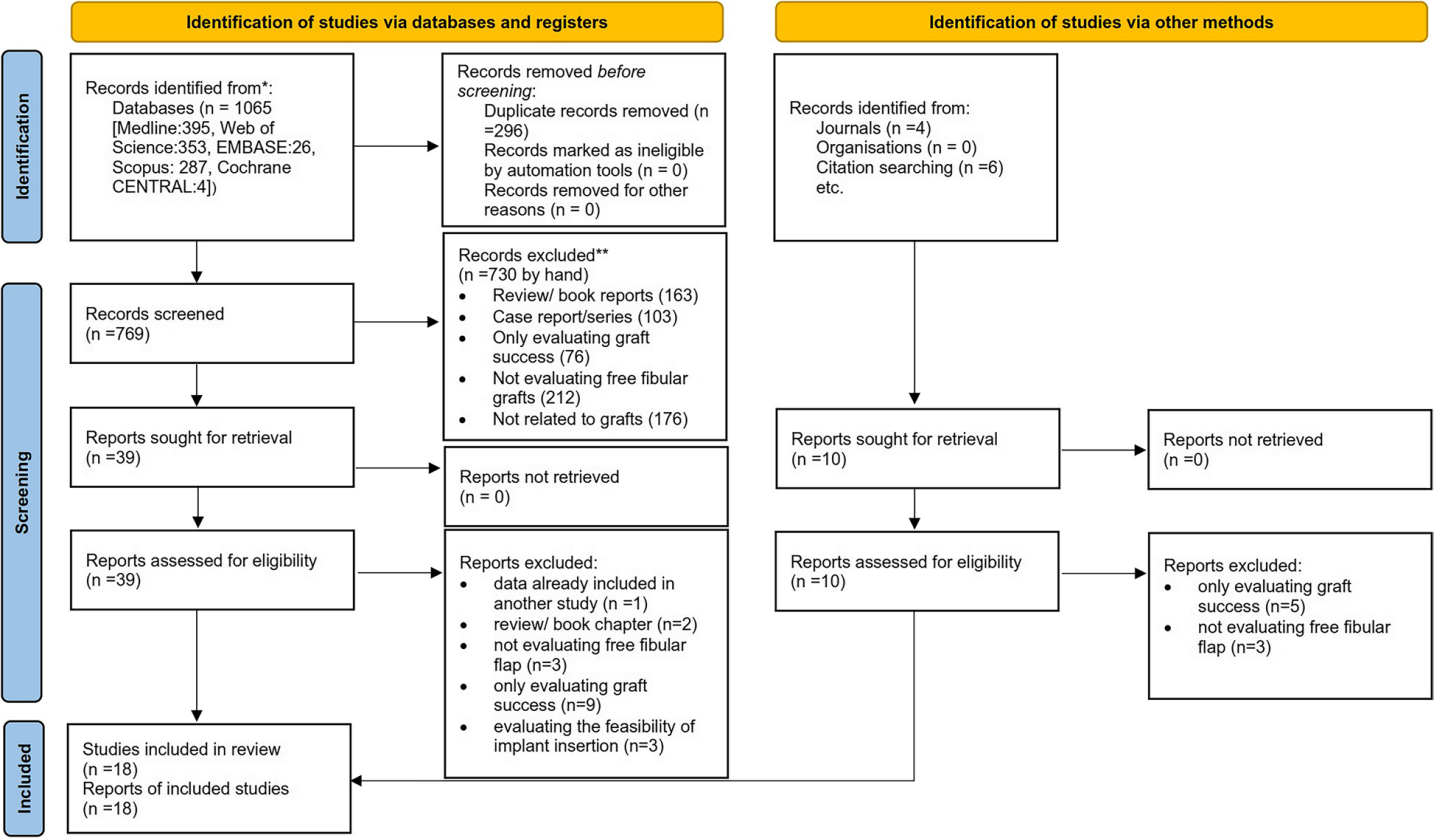
PRISMA 2020 flow diagram

After eliminating duplicate entries, 769 articles were identified through the search approach. A thorough assessment of titles and abstracts led to the exclusion of 730 articles, leaving 39 articles with potential relevance. Four studies emerged from the gray literature search, but only two met the criteria for inclusion. The 39 full-text articles from databases underwent a screening process based on predetermined inclusion and exclusion parameters. Upon examining the reference lists of these articles, six more studies were added. In the end, 18 studies met the criteria and were incorporated into the review, while 31 were dismissed after a full-text evaluation. A diagram illustrating the sequence of study identification, inclusion, exclusion, and the reasons for their exclusion can be found in Fig. 1.

### Study characteristics

The systematic review encompassed 18 studies, with 17 being retrospective [12–14, 16–28, 30] and one prospective cohort study [29]. In total, 774 patients (445 males, 252 females, and 77 unreported) were involved, and 1988 dental implants were used in free fibula grafts. These studies took place between 2006 and 2022, while patient treatments occurred from 1992 to 2019. Six studies focused solely on mandibular defects [18, 19, 23, 26, 27, 29], while the others examined both maxillary and mandibular resections. Additionally, four studies assessed implants placed in natural bones [12, 21, 25, 26], and three studies compared implant success rates across different graft types [14, 20, 30], such as scapula, DCIA, and MFC, alongside free fibular grafts.

Bone resections were performed due to head and neck neoplasia (both malignant and non-malignant), osteomyelitis, and osteoradionecrosis resulting from radiotherapy for malignant tumors. Sixteen studies evaluated implant success in free fibular grafts, while seven studies also measured the success rate of the grafts themselves [13, 16, 17, 22, 23, 25, 27]. Other outcomes, including patient-reported measures (function, comfort, esthetics) and factors affecting implant success rate (tobacco use, radiation before or after implant placement, age, and implant placement timing), were also documented in the studies. A summary of the study characteristics can be found in Table 2.

### Risk of bias

Upon evaluating the 18 studies using the NOS, the risk of bias was found to be diverse, with final assessment scores ranging from 5 to 9. The studies exhibited a mix of methodological quality, which should be taken into account when interpreting the results.

Out of the 18 studies, eight included control groups [12, 14, 20, 21, 25, 26, 28, 30], facilitating more robust comparisons and outcome evaluations. In contrast, some of the remaining 10 studies without control groups had limited generalizability and introduced bias into their results. Selection and recall biases were notably prevalent in some of the retrospective studies, especially those conducted by De Santis et al. and Parbo et al. [13, 23]. The risk-of-bias assessment for all the studies can be found in Table 3.

### Data synthesis

Meta-analyses were conducted to determine the success rates of implants and grafts, as well as the impact of smoking and radiotherapy on implant success. However, due to variations in intervention methods and outcome measures, meta-analyses for patient-reported outcomes, radiographical assessments, and the effects of malignancies and hyperbaric oxygen therapy (HBO) on implant success were not possible.

**Fig. 2:**
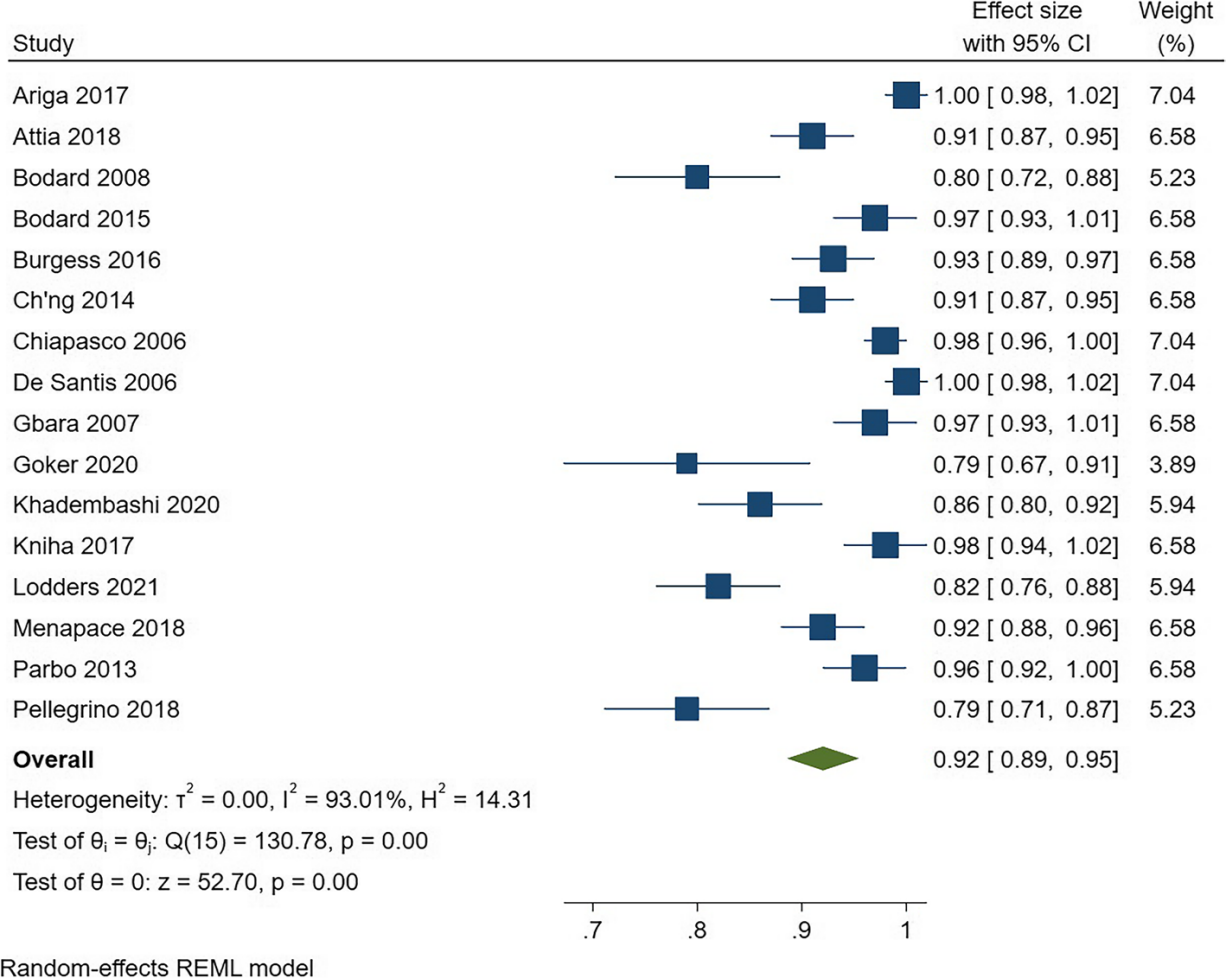
Forest plot for pooled success rate of implants in free fibula flap graft

**Fig. 3:**
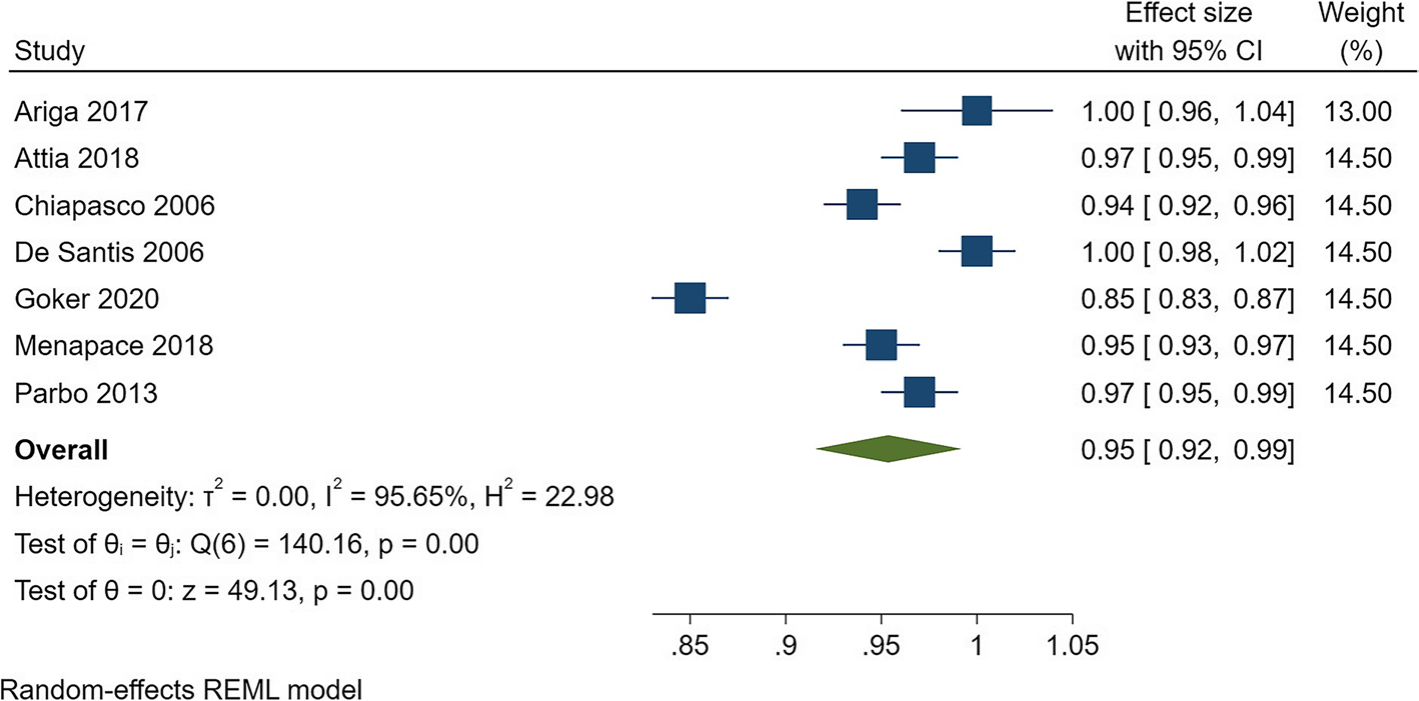
Forest plot for pooled success rate of free fibula flap grafts

Regarding implant success, the analysis included 16 studies and 1905 implantations in 745 free fibula grafts that were evaluated for at least 2 years. The pooled success rate was found to be 92% (*CI* = 0.89–0.95) but with significant heterogeneity (*I*^2^ = 93%) (Fig. 2). For graft success, the analysis of 174 grafts showed a success rate of 95% (*CI* = 0.92–0.99), but with high heterogeneity (*I*^2^ = 95%) (Fig. 3).

**Fig. 4:**
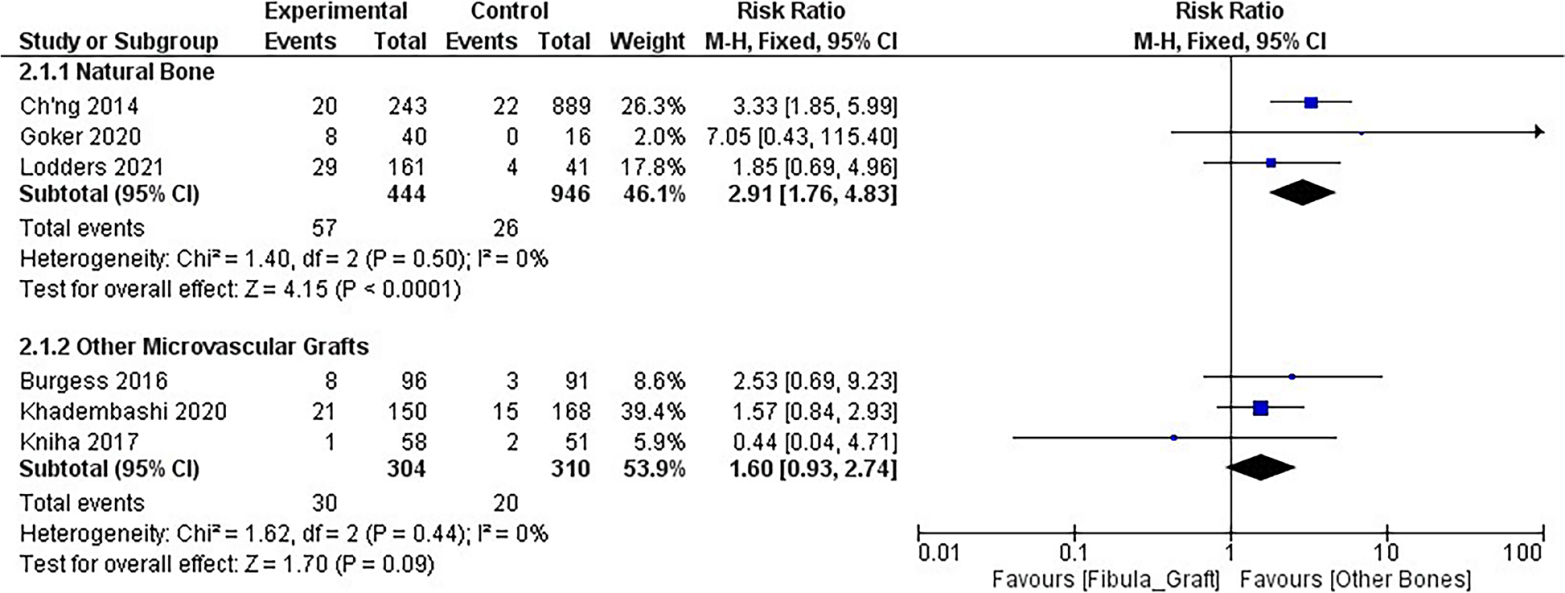
Forest plot for risk ratio of implant failure between fibula graft and natural bone/between fibula graft and other graft types

A comparison of the risk of implant failure in fibular grafts and other bones was also conducted, based on three studies and 1390 implants. The results showed that implants in fibular grafts have a 2.91 times higher failure rate than those in natural bones, which was statistically significant (*CI* = 1.76–4.83, *p* < 0.001). This analysis showed homogeneity in the results (*I*^2^ = 0%). However, when comparing the risk of implant failure in free fibular grafts with other grafts, no statistically significant difference was found (Fig. 4).

**Fig. 5:**
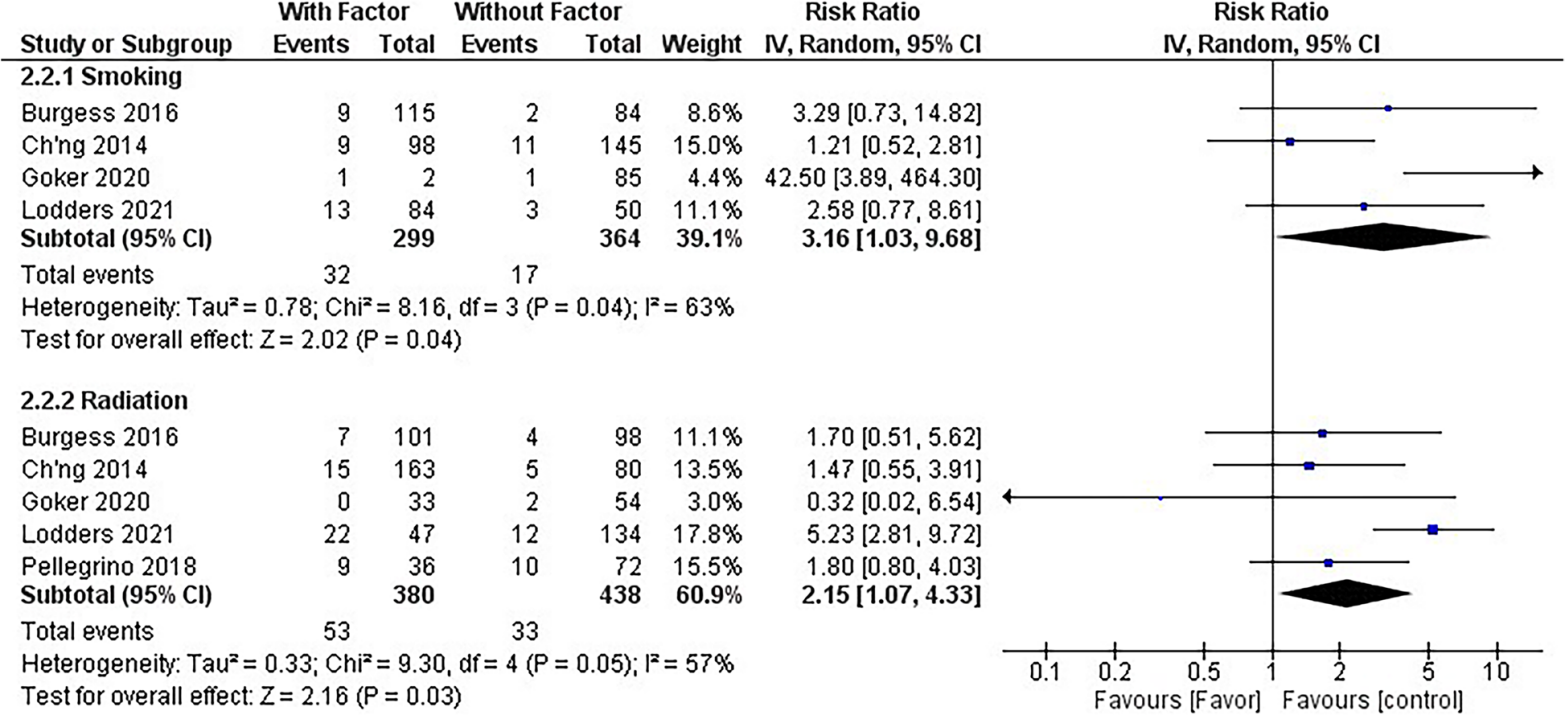
Forest plot for risk ratio of implant failure between the smoking and control group/radiotherapy and control group

Furthermore, a meta-analysis was conducted to examine factors influencing implant failure. The analysis of five studies with 818 implants (380 in radiated bone and 438 in surrounding healthy bone) showed that radiated bone had a 2.29 times higher risk of failure than unradiated bone, which was statistically significant (*CI* = 1.07–3.98, *p* = 0.03). Similarly, smokers had a 3.16 times (*CI* = 1.03–9.68, *p* = 0.04) higher risk of implant failure than nonsmokers, based on a comparison of 299 implants in smoking patients and 364 implants in non-smoking patients, which was also statistically significant (Fig. 5).

## Discussion

### Summary of results

The meta-analyses results determined that the pooled success rate for implants was 92% and for grafts, 95%, though both had significant heterogeneity. Implants in fibular grafts had a 2.91 times higher failure rate than those in natural bones, which was statistically significant. However, no significant difference was found when comparing free fibular grafts with other grafts. Additionally, radiated bone and smoking were identified as factors influencing implant failure, with radiated bone having a 2.29 times higher risk of failure and smokers having a 3.16 times higher risk compared to their respective counterparts.

Meta-analyses for patient-reported outcomes, radiographical assessments, and the effects of malignancies and HBO on implant success were not possible due to variations in methods and measures.

In the studies reviewed, various assessments were conducted to evaluate implant success, such as X-ray evaluations, bleeding on probing, and pocket depth measurements.

Attia et al.’s radiographic evaluation reported that 93 implants exhibited ≤ 1 mm of bone resorption, 11 had 1–2 mm, and 14 showed ≥ 3 mm. Their probing depth measurements revealed normal depths (1.0–4.0 mm) in 111 implants and 5.5 mm in 7 implants. Additionally, they found no sign of bleeding in 88 implants during the bleeding on probing assessment [17].

De Santis et al.’s X-ray evaluation indicated low bone resorption (1–2 mm below the head of the implant) after 1 year, although a specific percentage was not provided [23].

Gbara et al. observed that crestal bone resorption was less than 1 mm in 62 implants (53%), 1 to 2 mm in 35 implants (29.9%), and greater than 3 mm in 20 implants (17%). They reported no pathological probing depths in 93 of 121 implants, with depths ranging from 2 to 3 mm. Their sulcus bleeding index averaged 0.78, with 20 implants showing probing depths of 4 to 6 mm and an average sulcus bleeding index of 1.8. In 4 implants, probing depths exceeded 7 mm, with an average sulcus bleeding index of 3.5.

Pellegrino et al. reported bone resorption ranging from 0.5 to 8.1 mm (mean 2.2 ± 1 mm) at the 10-year follow-up, without providing a percentage. Their pocket depth measurements ranged from 2 to 9 mm, with a mean of 3.8 ± 2 mm [24].

These findings suggest that implant success in fibula free flaps is generally favorable, with minimal bone resorption, manageable probing depths, and limited bleeding on probing.

For patient-reported outcomes of functional rehabilitation after graft and implant placement, several studies have reported varying degrees of improvement in key areas such as dietary intake, mastication, speech, and esthetics. Ariga et al. observed improvements in dietary intake, mastication, and speech, along with high satisfaction regarding esthetics for the majority of their patients [16]. Similarly, Bodard et al. reported prosthesis satisfaction in most cases, with esthetic and dietary improvements observed more frequently in patients with fixed prostheses compared to removable dentures [19]. Furthermore, Lodders et al. found that functional dental rehabilitation was achieved for a majority of their patients, though patients with irradiated FFFs experienced slightly lower success rates [12].

Another study by Lodders et al. evaluated patient-reported scales for quality of life and functional rehabilitation, finding better emotional functioning, cognitive functioning, speech, mastication, speech, and reduced diarrhea for patients with implant dental rehabilitation (IDR) compared to those without [26]. Menapace et al. reported that patients in the primary implantation group experienced a shorter timeframe for nasogastric tube removal and had better oral competence and speech outcomes than those in the secondary implantation group [27]. However, these differences were not statistically significant and could be attributed to multiple factors. Overall, these studies indicate that functional rehabilitation outcomes can vary but generally show improvement in key areas such as dietary intake, mastication, speech, and esthetics following graft and implant placement.

### Factors influencing the success rate

The impact of smoking on implant success warrants further discussion, particularly when comparing active smokers and ex-smokers. Burgess et al. found that both ex-smokers and active smokers had lower survival rates (78% and 72%, respectively) compared to nonsmokers (94%) [20]. This suggests that even ex-smokers may be at a significantly higher risk of implant failure compared to nonsmokers which is similar to the results of graft success rate in the Chen et al. study [31]. Consequently, it is important to consider patients’ smoking history before graft and implant procedures and to provide additional interventions aimed at increasing implant success for these higher-risk individuals.

Furthermore, differences in the effects of radiation before and after implant placement should be considered. Ch'ng et al. demonstrated that preoperative radiation resulted in a lower survival rate (92%) compared to postoperative radiation (96.8%) [21]. Studies by Khadembashi et al. and Kniha et al. also found that pre-implant radiation significantly reduced implant success rates compared to those irradiated after implant placement [14, 30]. This is consistent with findings from other studies on implantation and radiotherapy in natural bone [32]. Some authors, such as Pompa et al. and Laverty et al., recommend implant insertion before radiation therapy to allow initial osseointegration to occur before irradiation, thus reducing the risk of late complications [33, 34]. Moreover, Lodders et al.’s study found that all the implants in actively smoking patients who underwent radiation failed, indicating that a combination of these risk factors may further exacerbate implant failure rates and emphasizing the need for careful patient selection and management in these cases [12].

Another aspect to consider is the difference in outcomes between primary and secondary reconstruction. Primary reconstruction refers to grafting performed immediately after the ablation of pathological bone, while secondary reconstruction occurs at a later time and in a separate surgery following the initial ablation. In the Chiapasco et al. study, no significant differences were found between these two methods in terms of implant success rates. However, it is important to note that the number of patients with secondary reconstruction in this study was relatively low (17), which may limit the conclusiveness of the findings [22].

The effects of various factors such as age, sex, alcohol consumption, and diabetes on implant success rates should also be considered. Khadembashi et al. found that increasing age and male gender increased the risk of implant failure, while Ch'ng et al. discovered that the success rate of implants in patients over 65 years of age was lower, although not statistically significant [14, 21]. Studies on implant outcomes in native bone have also reported mixed findings regarding the impact of age on implant failure [35]. Regarding alcohol consumption, Lodder et al. found no statistically significant differences between alcohol consumption and implant or graft failure. As for diabetes, Ch'ng et al. observed that in patients with controlled diabetes, there were no significant differences between the implant success rates of diabetic (96%) and nondiabetic patients (97%) [21].

HBO has been proposed as a potential method for improving implant success rates, particularly in patients with compromised healing conditions [36]. HBO involves the administration of 100% oxygen at pressures greater than atmospheric pressure, typically between 2 and 2.5 atmospheres absolute. This treatment increases the amount of dissolved oxygen in the bloodstream, which can enhance tissue oxygenation, reduce edema, and promote angiogenesis [37]. These physiological effects may contribute to improved healing and, consequently, increased implant success rates.

Several studies have reported positive outcomes when using HBO as an adjunctive therapy in dental implant procedures, particularly in patients who have undergone radiotherapy or have other risk factors that impair healing. HBO has been shown to improve bone quality, soft tissue healing, and implant osseointegration in these patients, leading to better overall success rates [38, 39]. Furthermore, HBO may reduce the risk of osteoradionecrosis in patients who have undergone radiotherapy. In our review study, two studies by Lodders et al. and Parbo et al. utilized HBO in all of their patients who had undergone radiotherapy [12, 13]. Despite the use of HBO therapy, these studies still found significant differences in the success rates between radiated and non-radiated implants. This observation suggests that HBO might not be as essential in implant success rates as some other studies claim. However, since all the radiated patients in these studies underwent HBO therapy, it is impossible to fully assess the effects of this treatment on radiated free fibula graft patients.

Nonetheless, HBO therapy could still be suggested as a possible intervention for patients with risk factors, particularly those who have undergone radiotherapy. It is important to note that the results from these studies should not be taken as definitive evidence against the benefits of HBO therapy but rather an indication that further research is needed.

In our review and meta-analysis, all the included studies had a follow-up period of at least two years after loading the implants. A notable observation across these studies was the considerable drop in implant success rates over time. For example, in the study by Khadembaschi et al., the success rate of FFF implants was 93% at 1-year, 90% at 2-year, 86% at 5-year, 83% at 7-year, and 69% at 9-year follow-up [14]. Similarly, in the Pellegrino et al. study, the success rate for FFF implants was 97.2% at 12-month follow-up, 86.5% at 60 months, and 79.3% at 120 months [28]. This decline in success rates highlights the importance of long-term follow-up when evaluating the effectiveness of dental implant procedures in free fibula graft patients.

The drop in success rates could be attributed to various factors such as aging, changes in health status, or the long-term effects of radiotherapy, which might impact the osseointegration and stability of the implants. Given these findings, it is crucial for future studies to consider the significance of long-term follow-up when assessing the success of dental implants in free fibula graft patients. Consistent monitoring and reporting of implant success rates at different stages of the follow-up period can help identify potential challenges and develop appropriate interventions to address them.

And finally, in the prospective study by Zweifel et al., the authors investigated the precision of simultaneous guided dental implantation in microvascular fibular flap reconstructions with and without additional guiding splints [29]. The study involved two groups: a trial group using additional tooth-borne and plate-borne splints for implant position and angulation verification and a control group following the standard preplanning protocol without additional splints. With a total of 8 patients, the results revealed that the average positioning error at the bone level was lower in the trial group (0.9 mm) than in the control group (1.3 mm). Similarly, the angulation errors in both buccolingual and axial planes were generally lower in the trial group. The use of intraoral and/or extraoral verification splints proved effective, with minimal additional operating room time required. This study underscores the potential benefits of employing additional guiding splints in dental implantation procedures for microvascular fibular flap reconstructions.

### Comparison with similar studies

There are other systematic reviews and meta-analysis to evaluate the success rate of implants in free fibular grafts. For example, Gangwani et al. assessed the success rate of implants in 10 retrospective studies [40]. Gangwani et al.’s study reported a 94% success rate (*CI* = 0.91 to 0.96) with an annual implant failure rate of 0.02 (*CI* = 0.01 to 0.03). Our study’s pooled success rate was 92% (*CI* = 0.89–0.95) for implant success and 95% (*CI* = 0.92–0.99) for graft success. We also investigated factors influencing implant failure, such as radiotherapy and smoking, which Gangwani’s study did not address.

Our study, which included 18 studies with 16 of them being part of the meta-analysis, provides a more comprehensive analysis compared to the systematic review and meta-analysis by Gangwani et al., which consisted of 10 studies. Our analysis evaluated not only the success rate of osseointegrated dental implants placed in fibula free flaps but also the factors affecting the success rate. In contrast, Gangwani et al. focused solely on the success rate of dental implants in fibula free flaps using the Albrektsson and colleagues’ criteria.

Furthermore, study done by Ardisson et al. focused on the implant success rate after mandible reconstruction with vascularized fibula bone grafts [41]. Their systematic review included 13 cohort studies which reported a success rate of approximately 98% for fibular reconstructions and 92.6% for implants placed in vascularized fibular grafts after a mean follow-up period of 40 months. They also observed that implant survival in irradiated patients was lower compared to nonirradiated patients, but alcohol and tobacco use showed no significant association with implant failure.

Our success rate results are closely aligned with those from the Ardisson et al. study, which reported a 95% graft success rate and a 92% implant success rate. Both studies found that radiotherapy negatively impacted the implant success rate. However, our study identified a significant difference in implant failure due to tobacco use, whereas Ardisson et al.’s study did not.

It is important to emphasize that Ardisson et al.’s study did not conduct a meta-analysis to evaluate the effects of tobacco and radiation on implant failure. Instead, they relied on a review of individual studies. In contrast, our study utilized a meta-analysis approach to assess these factors, providing a more rigorous and reliable assessment. Furthermore, our study included a larger number of studies (18), which adds to the robustness and reliability of our findings. Consequently, our study offers a more dependable evaluation of factors influencing implant success, including tobacco use and radiation exposure.

### Limitations and suggestions for further research

#### Limitations

High heterogeneity was observed in the meta-analyses for both implant and graft success rates, which could affect the reliability of the pooled success rates.

The majority of the studies were retrospective, potentially introducing biases such as selection and recall biases.

Due to variations in intervention methods and outcome measures, some meta-analyses (e.g., for patient-reported outcomes and radiographical assessments) were not possible, limiting the comprehensiveness of the results.

#### Suggestions for further research

Future studies should focus on conducting prospective, controlled trials to reduce biases and improve the quality of evidence in this area.

Standardization of outcome measures and intervention methods would facilitate more meaningful comparisons and enable more comprehensive meta-analyses.

Researchers should investigate the long-term success rates of implants and grafts in different patient populations, considering factors such as age, smoking history, and radiation therapy status.

Further research should explore the relationship between different implant and graft types and functional rehabilitation outcomes, such as dietary intake, mastication, speech, and esthetics, to inform clinicians on the best course of action for each patient.

Studies should examine the effectiveness of interventions aimed at increasing implant success rates in higher-risk individuals, such as smokers and those with a history of radiation therapy.

## Conclusion

In conclusion, this review and meta-analysis showed the success rates of dental implants in free fibula grafts, with pooled success rates of 92% for implants and 95% for grafts. The results suggest that implant success in fibula free flaps is generally favorable, with minimal bone resorption, manageable probing depths, and limited bleeding on probing. Patient-reported outcomes indicate improvements in key areas such as dietary intake, mastication, speech, and esthetics following graft and implant placement.

Several factors were identified as influencing implant success, including smoking, radiated bone, age, and gender. It is crucial to consider these factors when selecting patients for graft and implant procedures and to provide additional interventions aimed at increasing implant success for higher-risk individuals. The timing of radiation therapy, primary vs. secondary reconstruction, and the use of HBO therapy were also found to impact implant success rates, warranting further investigation.

A decline in success rates over time highlights the importance of long-term follow-up when evaluating dental implant effectiveness in free fibula graft patients. Consistent monitoring and reporting of implant success rates at different stages of the follow-up period can help identify potential challenges and develop appropriate interventions to address them.

## Data Availability

The datasets used and/or analyzed during the current study are available from the corresponding author on reasonable request.

## Abbreviations

FFF: Free fibula flap
PRISMA: Preferred Reporting Items for Systematic Reviews and Meta-Analyses
NOS: Newcastle-Ottawa scale
HBO: Hyperbaric oxygen therapy

## References

[1] Bahri R, Bhandari S. Maxillofacial defects: impact on psychology and esthetics. 2021.

[2] Mothopi MM, Owen CP, Howes DG, Naidoo LM (2012). The need for versatility in the prosthodontic treatment of maxillofacial defects. SADJ.

[3] Papageorgiou SN, Papageorgiou PN, Deschner J, Götz W (2016). Comparative effectiveness of natural and synthetic bone grafts in oral and maxillofacial surgery prior to insertion of dental implants: systematic review and network meta-analysis of parallel and cluster randomized controlled trials. J Dent.

[4] Haugen HJ, Lyngstadaas SP, Rossi F, Perale G (2019). Bone grafts: which is the ideal biomaterial?. J Clin Periodontol.

[5] Badhey AK, Khan MN (2020). Palatomaxillary reconstruction: fibula or scapula. Semin Plast Surg.

[6] Cho AM, Lopez J, Teven CM, Pourtaheri N, Do NTK, Jazayeri HE (2022). Outcomes in pediatric maxillofacial reconstruction with vascularized fibular flaps: a systematic review. J Craniofac Surg.

[7] Mandpe AH, Singer MI, Kaplan MJ, Greene D (1998). Alloplastic and microvascular restoration of the mandible: a comparison study. Laryngoscope.

[8] Garcia Blanco M, Ostrosky MA (2013). Implant prosthetic rehabilitation with a free fibula flap and interpositional bone grafting after a mandibulectomy: a clinical report. J Prosthet Dent.

[9] Zebolsky AL, Patel N, Heaton CM, Park AM, Seth R, Knott PD (2021). Patient-reported aesthetic and psychosocial outcomes after microvascular reconstruction for head and neck cancer. JAMA Otolaryngol Head Neck Surg.

[10] Attia S, Wiltfang J, Streckbein P, Wilbrand JF, El Khassawna T, Mausbach K (2019). Functional and aesthetic treatment outcomes after immediate jaw reconstruction using a fibula flap and dental implants. J Craniomaxillofac Surg.

[11] Bouchet B, Raoul G, Julieron B, Wojcik T (2018). Functional and morphologic outcomes of CAD/CAM-assisted versus conventional microvascular fibular free flap reconstruction of the mandible: a retrospective study of 25 cases. J Stomatol Oral Maxillofac Surg.

[12] Lodders JN, Leusink FKJ, Ridwan-Pramana A, Winters HAH, Karagozoglu KH, Dekker H (2021). Long-term outcomes of implant-based dental rehabilitation in head and neck cancer patients after reconstruction with the free vascularized fibula flap. J Craniofac Surg.

[13] Parbo N, Murra NT, Andersen K, Buhl J, Kiil B, Nørholt SE (2013). Outcome of partial mandibular reconstruction with fibula grafts and implant-supported prostheses. Int J Oral Maxillofac Surg.

[14] Khadembaschi D, Russell P, Beech N, Batstone MD (2021). Osseointegrated implant survival, success and prosthodontic outcomes in composite free flaps: a 10-year retrospective cohort study. Clin Oral Implants Res.

[15] Page MJ, McKenzie JE, Bossuyt PM, Boutron I, Hoffmann TC, Mulrow CD (2021). The PRISMA 2020 statement: an updated guideline for reporting systematic reviews. BMJ.

[16] Ariga P, Narayanan V, Jain AR, Philip JM, Nathan S (2017). Clinical and functional outcomes of implant prostheses in fibula free flaps. World J Dent.

[17] Attia S, Wiltfang J, Pons-Kühnemann J, Wilbrand JF, Streckbein P, Kähling C (2018). Survival of dental implants placed in vascularised fibula free flaps after jaw reconstruction. J Craniomaxillofac Surg.

[18] Bodard AG, Bemer J, Gourmet R, Lucas R, Coroller J, Salino S (2008). Dental implants and microvascular free fibula flap: 23 patients. Revue De Stomatologie De Chirurgie Maxillo-Faciale Et De Chirurgie Orale.

[19] Bodard AG, Salino S, Desoutter A, Deneuve S (2015). Assessment of functional improvement with implant-supported prosthetic rehabilitation after mandibular reconstruction with a microvascular free fibula flap: a study of 25 patients. JPD.

[20] Burgess M, Leung M, Chellapah A, Clark JR, Batstone MD (2017). Osseointegrated implants into a variety of composite free flaps: a comparative analysis. Head Neck.

[21] Ch'ng S, Skoracki RJ, Selber JC, Yu PR, Martin JW, Hofstede TM (2016). Osseointegrated implant-based dental rehabilitation in head and neck reconstruction patients. HEAD NECK-J SCI SPEC.

[22] Chiapasco M, Biglioli F, Autelitano L, Romeo E, Brusati R (2006). Clinical outcome of dental implants placed in fibula-free flaps used for the reconstruction of maxillo-mandibular defects following ablation for tumors or osteoradionecrosis. Clin Oral Implants Res.

[23] De Santis G, Pinelli M, Baccarani A, Pedone A, Spaggiari A, Jacob V (2006). Clinical and instrumental evaluation of implant stability after free fibula flaps for jaw reconstruction. Eur J Plast Surg.

[24] Gbara A, Darwich K, Li L, Schmelzle R, Blake F (2007). Long-term results of jaw reconstruction with microsurgical fibula grafts and dental implants. Int J Oral Maxillofac Surg.

[25] Goker F, Baj A, Bolzoni AR, Maiorana C, Giannì AB, Del Fabbro M (2020). Dental implant-based oral rehabilitation in patients reconstructed with free fibula flaps: clinical study with a follow-up 3 to 6 years. Clin Implant Dent Relat Res.

[26] Lodders JN, van Baar GJC, Vergeer MR, Jansen F, Schulten EAJM, Lissenberg-Witte BI (2022). Implant-based dental rehabilitation in head and neck cancer patients after maxillofacial reconstruction with a free vascularized fibula flap: the effect on health-related quality of life. SCC.

[27] Menapace DC, Van Abel KM, Jackson RS, Moore EJ (2018). Primary vs secondary endosseous implantation after fibular free tissue reconstruction of the mandible for osteoradionecrosis. Jama Fac Plast Surg.

[28] Pellegrino G, Tarsitano A, Ferri A, Corinaldesi G, Bianchi A, Marchetti C (2018). Long-term results of osseointegrated implant-based dental rehabilitation in oncology patients reconstructed with a fibula free flap. Clin Implant Dent Relat Res.

[29] Zweifel D, Bredell MG, Lanzer M, Rostetter C, Rücker M, Studer S (2019). Precision of simultaneous guided dental implantation in microvascular fibular flap reconstructions with and without additional guiding splints. J Oral Maxillofac Surg.

[30] Kniha K, Möhlhenrich SC, Foldenauer AC, Peters F, Ayoub N, Goloborodko E (2017). Evaluation of bone resorption in fibula and deep circumflex iliac artery flaps following dental implantation: a three-year follow-up study. J Craniomaxillofac Surg.

[31] Chen YM, Wu JL, Gokavarapu S, Shen QC, Ji T (2017). Radiotherapy and smoking history are significant independent predictors for osteosynthesis-associated late complications in vascular free fibula reconstruction of mandible. J Craniofac Surg.

[32] Shugaa-Addin B, Al-Shamiri HM, Al-Maweri S, Tarakji B (2016). The effect of radiotherapy on survival of dental implants in head and neck cancer patients. J Clin Exp Dent.

[33] Pompa G, Saccucci M, Di Carlo G, Brauner E, Valentini V, Di Carlo S (2015). Survival of dental implants in patients with oral cancer treated by surgery and radiotherapy: a retrospective study. BMC Oral Health.

[34] Laverty DP, Addison O, Wubie BA, Heo G, Parmar S, Martin T (2019). Outcomes of implant-based oral rehabilitation in head and neck oncology patients-a retrospective evaluation of a large, single regional service cohort. Int J Implant Dent.

[35] Thiebot N, Hamdani A, Blanchet F, Dame M, Tawfik S, Mbapou E (2022). Implant failure rate and the prevalence of associated risk factors: a 6-year retrospective observational survey. J Oral Med Oral Surg.

[36] Curi MM, Oliveira dos Santos M, Feher O, Faria JCM, Rodrigues ML, Kowalski LP (2007). Management of extensive osteoradionecrosis of the mandible with radical resection and immediate microvascular reconstruction. J Oral Maxillofac Surg Med Pathol.

[37] Kelishadi SS, St.-Hilaire H, Rodriguez ED.  (2009). Is simultaneous surgical management of advanced craniofacial osteoradionecrosis cost-effective?. Plast Reconstr Surg.

[38] Coulthard P, Patel S, Grusovin GM, Worthington HV, Esposito M (2008). Hyperbaric oxygen therapy for irradiated patients who require dental implants: a Cochrane review of randomised clinical trials. Eur J Oral Implantol.

[39] Shah DN, Chauhan CJ, Solanki JS (2017). Effectiveness of hyperbaric oxygen therapy in irradiated maxillofacial dental implant patients: a systematic review with meta-analysis. J Indian Prosthodont Soc.

[40] Gangwani P, Almana M, Barmak B, Kolokythas A (2022). What is the success of implants placed in fibula flap? a systematic review and meta-analysis. J Oral Maxillofac Res.

[41] Ardisson A, Senna P, Granato R, Bergamo E, Bonfante E, Marin C (2022). Success rate of mandible implants placed in vascularized fibula bone graft: a systematic review. J Oral Implantol.

